# Generalised cost-effectiveness analysis of 159 health interventions for the Ethiopian essential health service package

**DOI:** 10.1186/s12962-020-00255-3

**Published:** 2021-01-06

**Authors:** Getachew Teshome Eregata, Alemayehu Hailu, Karin Stenberg, Kjell Arne Johansson, Ole Frithjof Norheim, Melanie Y. Bertram

**Affiliations:** 1 Bergen Center for Ethics and Priority Setting, University of Bergen, Bergen, Norway; 2 Ministry of Health of Ethiopia, Addis Ababa, Ethiopia; 3 Department of Health Systems Governance and Financing, World Health Organization, Geneva, Switzerland; 4 Harvard T. H. Chan School of Public Health, Boston, USA

**Keywords:** Cost effectiveness analysis, Health care rationing, Priority setting, Ethiopia, Essential health services

## Abstract

**Background:**

Cost effectiveness was a criterion used to revise Ethiopia’s essential health service package (EHSP) in 2019. However, there are few cost-effectiveness studies from Ethiopia or directly transferable evidence from other low-income countries to inform a comprehensive revision of the Ethiopian EHSP. Therefore, this paper reports average cost-effectiveness ratios (ACERs) of 159 health interventions used in the revision of Ethiopia’s EHSP.

**Methods:**

In this study, we estimate ACERs for 77 interventions on reproductive maternal neonatal and child health (RMNCH), infectious diseases and water sanitation and hygiene as well as for 82 interventions on non-communicable diseases. We used the standardised World Health Organization (WHO) CHOosing Interventions that are cost effective methodology (CHOICE) for generalised cost-effectiveness analysis. The health benefits of interventions were determined using a population state-transition model, which simulates the Ethiopian population, accounting for births, deaths and disease epidemiology. Healthy life years (HLYs) gained was employed as a measure of health benefits. We estimated the economic costs of interventions from the health system perspective, including programme overhead and training costs. We used the Spectrum generalised cost-effectiveness analysis tool for data analysis. We did not explicitly apply cost-effectiveness thresholds, but we used US$100 and $1000 as references to summarise and present the ACER results.

**Results:**

We found ACERs ranging from less than US$1 per HLY gained (for family planning) to about US$48,000 per HLY gained (for treatment of stage 4 colorectal cancer). In general, 75% of the interventions evaluated had ACERs of less than US$1000 per HLY gained. The vast majority (95%) of RMNCH and infectious disease interventions had an ACER of less than US$1000 per HLY while almost half (44%) of non-communicable disease interventions had an ACER greater than US$1000 per HLY.

**Conclusion:**

The present study shows that several potential cost-effective interventions are available that could substantially reduce Ethiopia’s disease burden if scaled up. The use of the World Health Organization’s generalised cost-effectiveness analysis tool allowed us to rapidly calculate country-specific cost-effectiveness analysis values for 159 health interventions under consideration for Ethiopia’s EHSP.

## Background

Priority setting allows consensus to be reached on which interventions to include in an essential health service package (EHSP), on which interventions to scale up first and on which intervention to scale down [1–3]. In this process, various approaches can be applied to compare interventions, of which cost effectiveness is the most widely used globally [4]. Cost-effectiveness analysis (CEA) plays a central role in decision making in many health technology assessment agencies in high-income countries [5], and there has recently been a growing interest in using cost effectiveness in defining national EHSPs in low- and middle-income countries [6].

In the Ethiopian EHSP revision, cost effectiveness was a criterion chosen to compare health interventions in terms of value for money [7], but there are only a few CEAs of health interventions from Ethiopia and other low-income countries. For instance, Hailu et al. examine the cost effectiveness of malaria prevention interventions [8], Memirie et al. examine that of maternal and neonatal interventions [9], Strand et al. evaluate that of neuropsychiatric services [10], and Tolla et al. examine that of cardiovascular disease prevention and treatment interventions [11].

Most of those studies applied an incremental cost-effectiveness approach that compares the cost effectiveness of adding new interventions against the current practice in the area [12]. This approach assumes that the current practice is organised in the most efficient way possible and thus does not account for existing inefficiencies in the health system. With incremental/marginal analysis, it is difficult to examine whether the current mix of interventions represents an efficient use of resources [12, 13]. Although these pieces of evidence are vital in informing the setting of priorities in decision making in specific sub-programme areas or for specific diseases, particularly when the existing package is assumed to allocate efficiently, they are less relevant in informing the sector-wide analysis of EHSP revisions [14, 15].

Therefore, the World Health Organization (WHO), in its CHOosing Interventions that are Cost Effective (CHOICE) programme, proposes a generalised CEA that compares all interventions with ‘doing nothing’ or a ‘null scenario’ [13]. This approach assesses whether the current mix of interventions is efficient and whether a proposed new technology or intervention is appropriate. It also provides decision makers with information on what they could achieve if they reallocated resources in the most efficient way. This approach provides broader generalisability of the CEA results and is considered an appropriate method for redefining an EHSP [13]. Therefore, this paper uses the WHO-CHOICE tool to calculate an ACER for the 159 relevant health interventions for use in the revision of Ethiopia’s EHSP.

## Methods

### Study population and context

This study was conducted in Ethiopia in 2019 as part of the revision of the country’s EHSP [7]. Ethiopia has a large disease burden, with average life expectancy of 65.5 [16, 17]. Communicable, maternal, neonatal, and nutritional disorders (CMNNDs) represent the greatest disease burden, accounting for 58% of disability-adjusted life year (DALY) loss in 2017. In the same year, the burden of NCDs, such as cardiovascular diseases, diabetes and cancer, accounted for 34% of the burden. About 8% of the DALYs were from emergencies and injuries [17]. Furthermore, Ethiopia is a low-income country, with a Gross Domestic Product (GDP) per capita of US$953 in 2019 [18] and a per capita health expenditure of about US$33 in 2016/17 [19]. Further reduction or slow increment of the health expenditure is expected in Ethiopia because of the impact of COVID-19 pandemic on the economic growth of the country and its global impact. Therefore, it is crucial to invest limited resources efficiently.

### Interventions

A breakdown of interventions by the conditions they prevent or treat is provided in Table 1. A total of 1018 interventions were analysed for the EHSP. The current version of the WHO-CHOICE generalised cost-effectiveness analysis (GCEA) tool includes about 400 interventions [20], of which 159 were found to be relevant for the Ethiopian EHSP. We grouped the 159 interventions into 12 groups that matches with the sub-programme areas classification of intervention list in the EHSP. In general, and slightly over half of them fell under either reproductive, maternal, neonatal and child health (RMNCH) (28.3%), mental health (12.6%) or policies against NCDs (10.1%), such as physical inactivity, excessive alcohol use and tobacco, sugar and salt intake (Table 1).

**Table 1:** Frequency and proportion of interventions evaluated by sub-programme area, 2019

Intervention by sub-programme area	N	%	Spectrum impact model used
RMNCH	44	28.3	LiST, FamPlan
Mental health	20	12.6	NCD impact
Policy interventions on NCDs	16	10.1	NCD impact
Cervical cancer	13	8.2	NCD impact
Respiratory disease	12	7.6	NCD impact
Colorectal cancer	11	6.9	NCD impact
Breast cancer	10	6.3	NCD impact
Tuberculosis	10	6.3	TIME Estimates and TIME impact
Nutrition	9	5.7	LiST
HIV/AIDS	5	3.1	AIM and GOALS
Malaria	5	2.5	LiST
Water hygiene and sanitation	4	2.5	LiST
Total	159	100	

The level of detail varies across the sub-programme areas

### Health effects of the interventions

We used the WHO-CHOICE GCEA tool to analyse the country-level health benefits of each intervention [21]. This model examines for each disease of interest (by incidence, remission and case fatality rates) how proportions of the population transit between health states in the presence or absence of an intervention. The Global Burden of Disease disability weights were used to evaluate the health state in the time spent in each health state, and the health effects generated by each intervention are presented as healthy life years (HLYs) gained [22].

We applied various integrated impact-modelling modules of the latest version of Spectrum software to model the health benefits of each intervention [22] and applied the DemProj module to project population growth and other underlying demographic parameters (Table 1). This module uses World Population Prospects 2017 data from the United Nations Population Division. The FamPlan module was used to estimate the impact of family planning interventions. In this module, we used data from the 2016 Ethiopian Demographic Health Survey. We employed the AIDS Impact Module (AIM) (which was initially developed by UNAIDS to make national and regional HIV estimates every 2 years) to estimate the impact of interventions against HIV, and we employed the TIME Estimates and TIME impact Module to estimate the health impact of tuberculosis (TB) interventions. For RMNCH, nutrition and Water sanitation and hygiene (WASH) interventions, the Lives Saved Tool (LiST) module was employed, and we used the non-communicable disease impact module to calculate the impact of NCD policy interventions and other interventions against cancer and respiratory disease as well as mental health, neurological and substance use disorders [22].

The spectrum software includes default input for many countries based on data from various sources (i.e. systematic reviews, individual studies, national and regional reports, GBD etc.). We downloaded and used country-specific data for Ethiopia in the Spectrum software. The Country Data Package was prepopulated with the total population, population in need, target population, disease burden and effect size for each intervention. We carefully reviewed all the default input with programme area experts at the Ministry of Health, and appropriate changes were made when deemed necessary. A more detailed explanation of each of the intervention input assumptions is provided elsewhere [22, 23].

### Costs of interventions

The identification, measurement and valuation of the costs of all the interventions were conducted from the health system’s perspective, accounting for the full cost of delivering an intervention, regardless of who currently pays for it. The ingredients costing approach was used, in which each input of delivering the intervention is identified and the quantity of each resource required by the intervention is multiplied by the unit price of each input (i.e., the unit price × quantity approach was applied) [12]. In the WHO-CHOICE GCEA tool, all the ingredients, based on expert recommendations, are provided as default values, and the country team reviewed the inputs and made changes when necessary. For example, all the drugs and supplies needed to provide each service were systematically identified, accounting for the cost of delivering the drugs and supplies from the point of production or purchase to the point of use (i.e., the cost of transportation, storage, shipment and customs clearance). Default prices for drugs and suppliers within the GCEA tool are taken from an international drug price database (MSH). We updated the prices of some drugs and supplies based on data from the Ethiopian Pharmaceutical Supply Agency and the Logistics Department of the Ministry of Health. To account for the cost of delivering drugs and supplies, an average mark-up of 6% of the price was generally taken. For drugs needing a cold supply chain, an additional 13% of the cost of the drug was taken as mark-up as the cold-chain system incurs an additional cost. For Long-lasting Insecticidal Nets (LLINs), a 26% mark-up was taken as LLINs are relatively bulky and their transportation, loading and unloading incur an additional cost [24].

Health personnel costs for providing the interventions were also included. The salary scale of the health workforce, such as the salaries and benefits of nurses, doctors and pharmacists, was based on the most up-to-date data from the Human Resource Department of the Ministry of Health of Ethiopia. Staff time use was calculated on the assumption that, on average, each person works 8 h per day over 230 working days per year. Inpatient cost per day and outpatient cost per visit were taken from the WHO-CHOICE model [25].

Programme costs were also included in this analysis [24]. Programme costs are the non-health care delivery costs associated with delivering an intervention programme that are incurred at a level other than the intervention’s point of delivery. They include costs incurred at district, provincial or central levels and exclude costs incurred at facility or patient levels. They include the cost of administration and planning, media and communication, law enforcement, training, monitoring and evaluation. All costs were valued using 2019 US dollars (USDs). All cost input data originally collected in Ethiopian Birr (ETB) were first converted to USD using the average exchange rate for the year and were later converted to 2019 USD using the GDP deflator.

### Cost-effectiveness analysis

To account for the impact of an intervention in the long term (steady state), we followed in this cost-effectiveness analysis model a hypothetical Ethiopian population cohort over a 100-year time horizon starting in 2019. The average cost effectiveness of the intervention was computed as a ratio of the total cost of the intervention to total health life years (HLYs) gained from the intervention [12, 26]. The interventions were ranked and compared based on their ACERs. Both costs and health outcomes were discounted at an annual rate of 3% [13].

### Cost-effectiveness thresholds

A cost-effectiveness threshold (CET) is an explicit cut-off point for assessing the opportunity cost of interventions, with interventions having a cost-effectiveness ratio below the threshold being considered to offer good value for money [27]. There is a long-standing debate concerning the CET [18, 28, 29]. In the case of sector-wide analysis of health interventions using a GCEA, a CET is not required because the purpose of a GCEA is to compare the whole list of interventions against the comparator of doing nothing, and the ACERs of interventions should be compared with one another, even across programme areas, and not against a predefined CET [14, 15]. In this study, therefore, we did not apply a CET; instead, we report the ACERs in ascending order in bar graphs for each programme area. However, we use US$100 and US$1000 per HLY gained as references to summarise and present the ACER results.

## Results

In this study, we identified cost-effectiveness estimates for 159 interventions. An overview of the distribution of the ACERs is presented in Table 2. Of the total number of interventions evaluated in this study, 58 (37%) have an ACER of less than US$100 per HLY, 104 (65%) have an ACER of less than US$500 per HLY and 119 (75%) have an ACER of less than US$1000 per HLY gained.

**Table 2:** Summary of ACERs (USD per HLY) of the interventions by sub-programme areas

Sub-programme	Mean	SD	Median	p25	p75	Min	Max	% < $1000
HIV/AIDS	106	167	34	20	61	13	403	100
RMNCH	116	258	37	13	113	0.4	1591	97
WASH	122	219	16	9	234	5	451	100
Tuberculosis	143	12	139	137	147	129	163	100
Nutrition	262	312	72	37	580	31	746	100
Cervical cancer	870	1818	111	36	628	34	6534	77
Mental health	1045	1944	185	120	944	31	7610	75
Malaria	1163	1186	1310	79	1469	40	2915	40
NCD policy interventions	1834	2759	437	202	3053	26	9115	69
Breast cancer	2157	1895	1535	1032	2203	366	6104	20
Chronic respiratory diseases	2307	3344	809	368	1484	164	8856	50
Colorectal cancer	3920	1967	4646	2493	5436	783	5602	18
Overall	1014	1926	151	40	783	0.4	9115	75

SD: standard deviation; p25: 25th percentile; p75: 75th percentile; Min: minimum; Max: Maximum; % < $1000: proportions of interventions within that program area with ACERs lower than $1000 per HLY

Five interventions (basic palliative care for colorectal cancer, colorectal cancer treatment at stage 4, relapse prevention medication for alcohol use/dependence, inhaled short-acting beta-agonist for intermittent asthma and theophylline + high-dose inhaled beclometasone + short-acting beta-agonist for asthma) have an ACER above US$10,000 per HLY. Therefore, in the summary statistics provided in Table 2, we exclude these five interventions as they represent extreme values.

We estimated ACERs ranging from less than US$1 per HLY gained (for family planning) to about US$48,000 per HLY gained (for treatment of stage 4 colorectal cancer). A large majority (97%) of RMNCH and infectious disease interventions had an ACER of less than US$1000 per HLY, and a substantial proportion (44%) of NCD interventions had an ACER of greater than US$1000 per HLY (Table 2).

We present the full costs and effectiveness of all the interventions in supplement table (Additional file 1). Below, we present the key findings for major programme areas.

### Cost effectiveness of RMNCH interventions

**Fig. 1:**
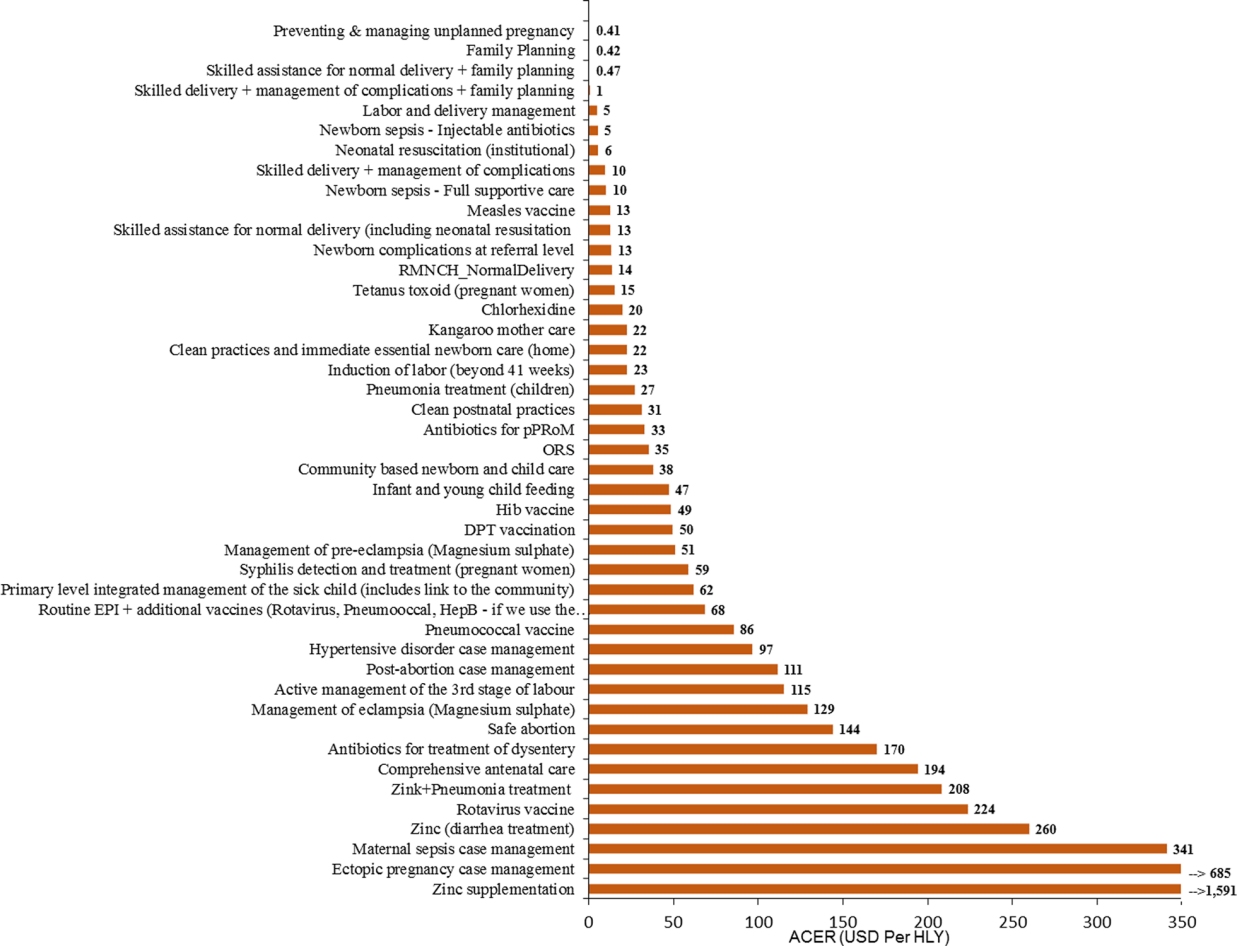
ACERs for RMNCH (Trimmed at US$350)

All RMNCH interventions except zinc supplementation (ACER = 1591 USD/HLY) and ectopic pregnancy case management (ACER = 685 USD/HLY) had an ACER of less than US$400 per HLY (Fig. 1). The three most cost-effective interventions in this category are preventing and managing unplanned pregnancy (ACER = 0.41 USD/HLY), provision of family planning services alone (ACER = 0.42 USD/HLY) and provision of skilled assistance for normal delivery, including postpartum family planning (ACER = 0.47 USD/HLY). All immunisation interventions cost less than US$100 (e.g., the Hib vaccine costs 49 USD/HLY, routine EPI + additional vaccines cost 68 USD/HLY and pneumococcal vaccine costs 86 USD/HLY).

### Cost effectiveness of HIV/AIDS, TB and malaria interventions

**Fig. 2:**
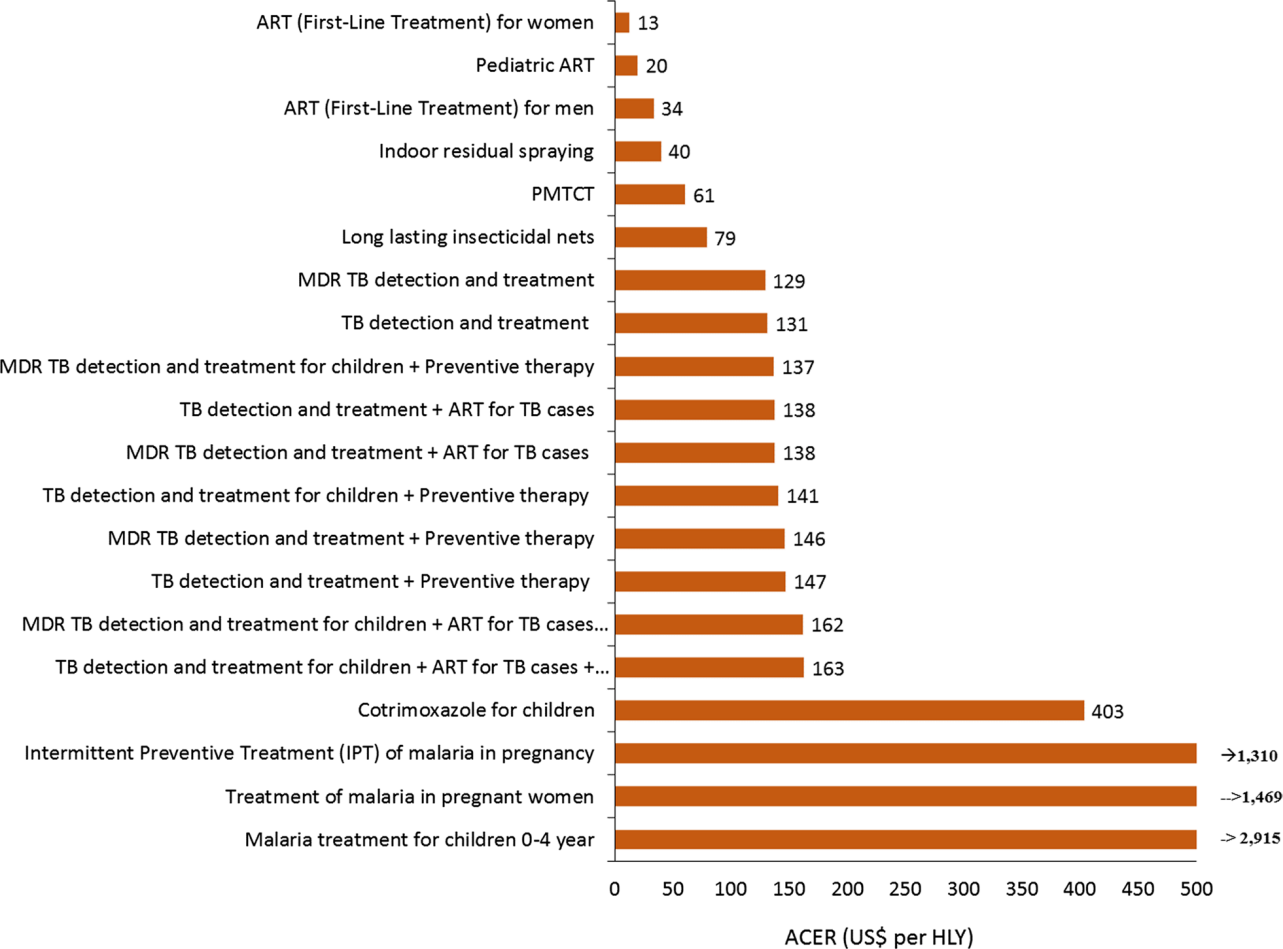
ACERs for HIV, TB and malaria interventions (Trimmed at US$500)

All the HIV/AIDS interventions had an ACER of less than US$100 per HLY gained except cotrimoxazole for children, which costs US$403 per HLY. Paediatric anti-retroviral therapy (ART) costs US$20 per HLY, Prevention of Mother to Child Transmission (PMTC) of HIV costs US$61 per HLY, ART for adult women costs US$13 per HLY and ART for adult men costs US$34 per HLY. In this study, we evaluated four anti-malaria interventions. While the use of insecticide-treated materials costs US$79 per HLY and indoor residual spraying costs US$40 per HLY, Intermittent Preventive Therapy (IPT) for pregnant women costs US$1310 per HLY and treatment of malaria for pregnant women costs US$1469 per HLY. The 10 TB interventions evaluated in this study have ACERs ranging from US$129 per HLY (for the detection and treatment of multidrug-resistance tuberculosis (MDR-TB) using a smear or culture) to US$163 per HLY (for the detection and treatment of TB using a combination of smear and Xpert) (Fig. 2).

**Fig. 3:**
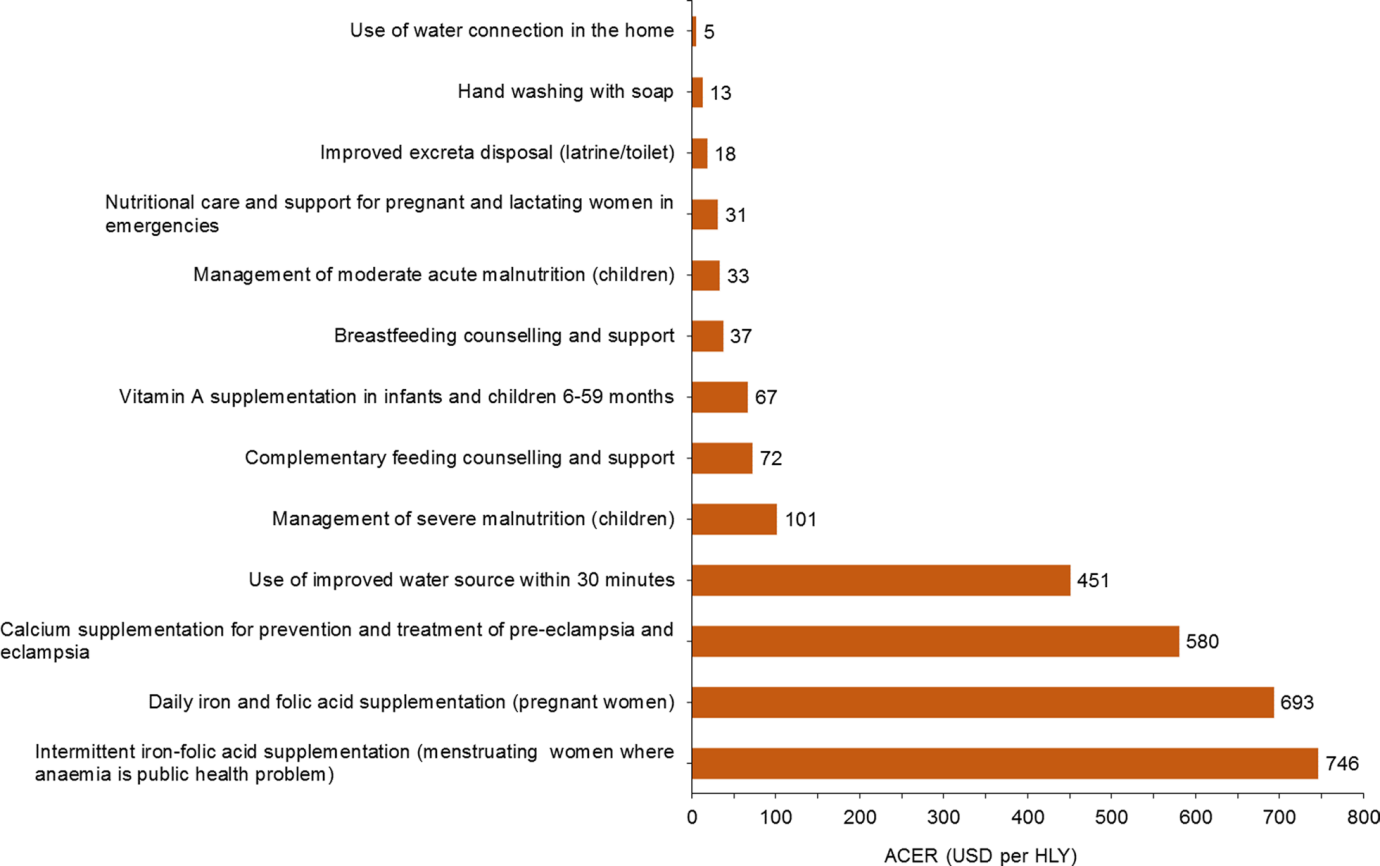
ACERs for nutrition and WASH interventions

Of the 13 WASH and nutrition interventions in this study, the three most cost-effective were use of a water connection in the home (ACER = US$5 per HLY), handwashing with soap (ACER = US$13 per HLY) and improved excreta disposal (latrine/toilet) (ACER = US$13 per HLY). Intermittent iron-folic acid supplementation for menstruating women where anaemia is a public health problem costs US$746 per HLY (Fig. 3).

### Cost effectiveness of NCD policy interventions

For the 16 policy interventions against NCDs evaluated in this study, the ACERs range from a high of US$9115 per HLY gained (for prevention of hazardous alcohol use using legal enforcement to restrict alcohol advertising) to a low of US$26 per HLY gained (for reduction of salt intake by harnessing/involving industries for reformulation). Most of the tobacco prevention interventions were very cost effective. For instance, the ACER for protecting people from tobacco smoke was US$232 per HLY while warning about the danger of tobacco using labels costs US$411 per HLY. The ACER for warning people about the danger of tobacco through mass media campaigns was US$515 per HLY gained, for enforcing bans on tobacco advertising US$105 per HLY gained and for enforcing youth access restrictions on tobacco US$1728 per HLY gained.

**Fig. 4:**
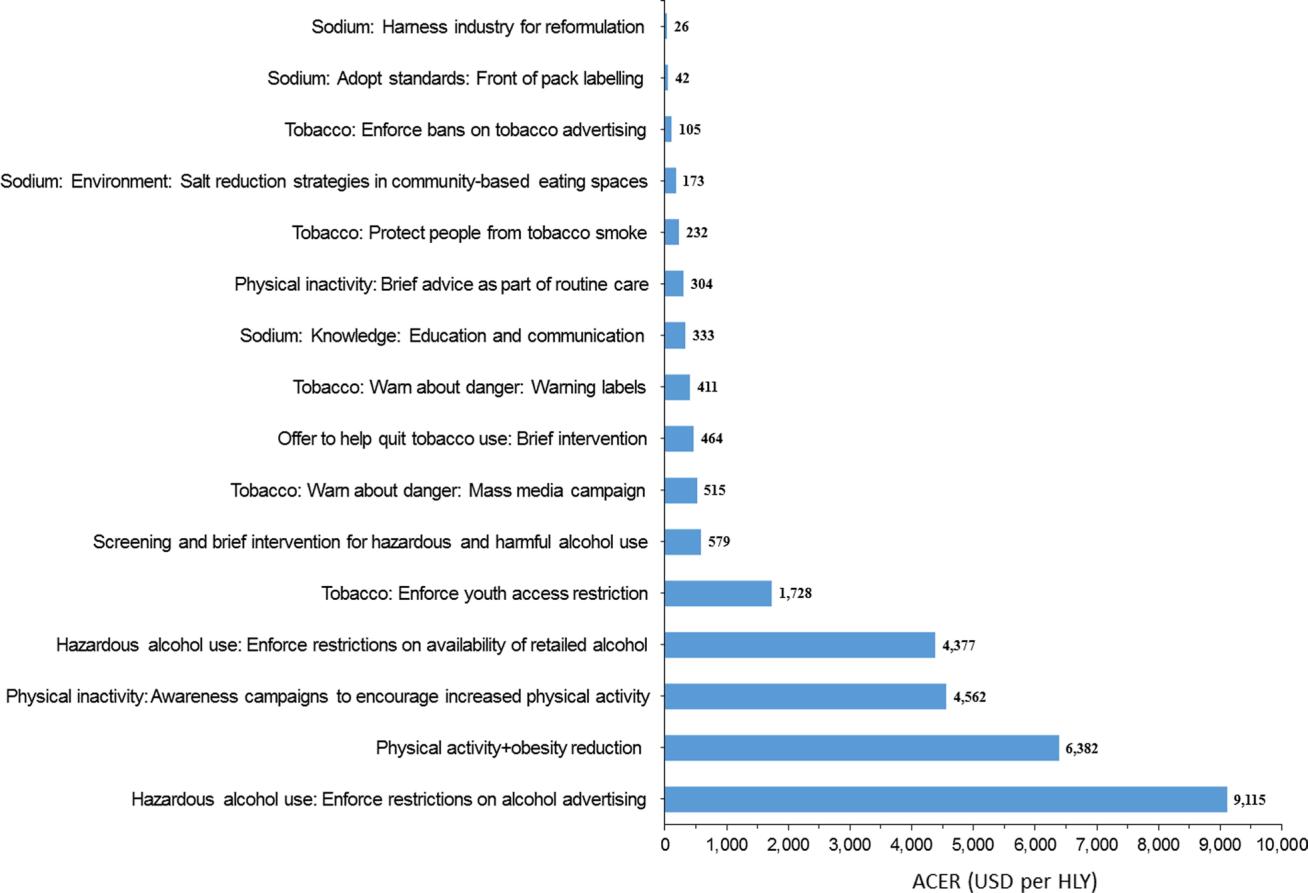
ACERs for NCD policy interventions

Intervention to enforce restrictions on the availability of retailed alcohol was US$4377 per HLY gained while screening and brief intervention for hazardous and harmful alcohol use was only US$579 per HLY gained. Most of the salt intake restriction interventions have the lowest cost-effectiveness ratios for Ethiopia. For instance, adopting standards in front-of-pack labelling costs US$42 per HLY. Providing education and communication costs US$333 per HLY, and pursuing salt reduction strategies in community-based eating spaces costs US$173 per HLY gained (Fig. 4).

### Cost effectiveness of cancer interventions

**Fig. 5:**
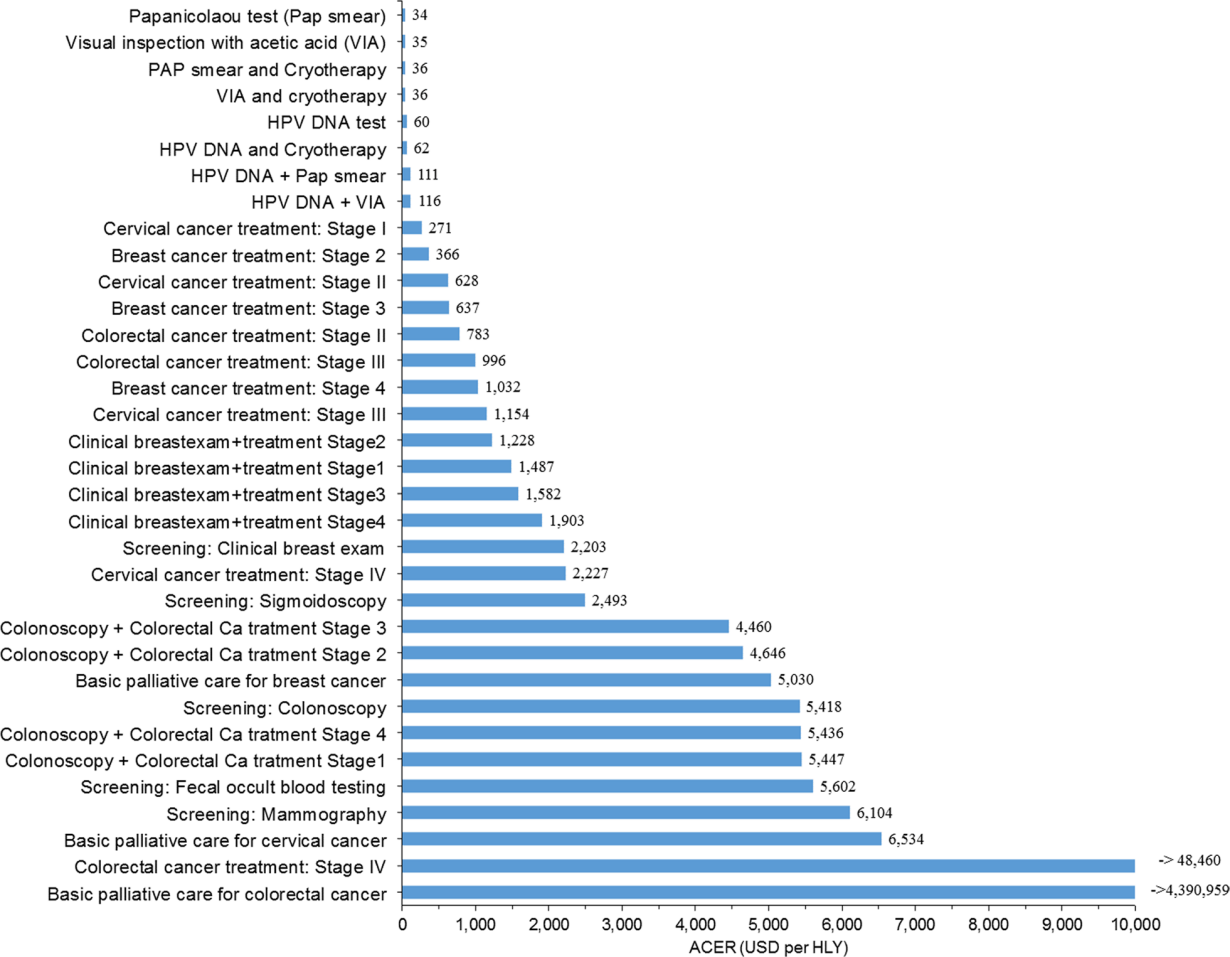
ACERs for cancer interventions (Trimmed at US$10,000)

All the early detection and screening interventions for cervical cancer cost less than US$100 per HLY. For example, a Papanicolaou test (Pap smear) costs US$34, visual inspection with acetic acid (VIA) costs US$35 and the HPV-DNA test costs US$60 per HLY gained. However, screening of breast cancer with clinical examination costs US$2203 per HLY and with mammography US$6104 per HLY. Similarly, colorectal cancer screening with sigmoidoscopy costs US$2493 and with colonoscopy US$5418 per HLY (Fig. 5).

### Cost effectiveness of mental health interventions

**Fig. 6:**
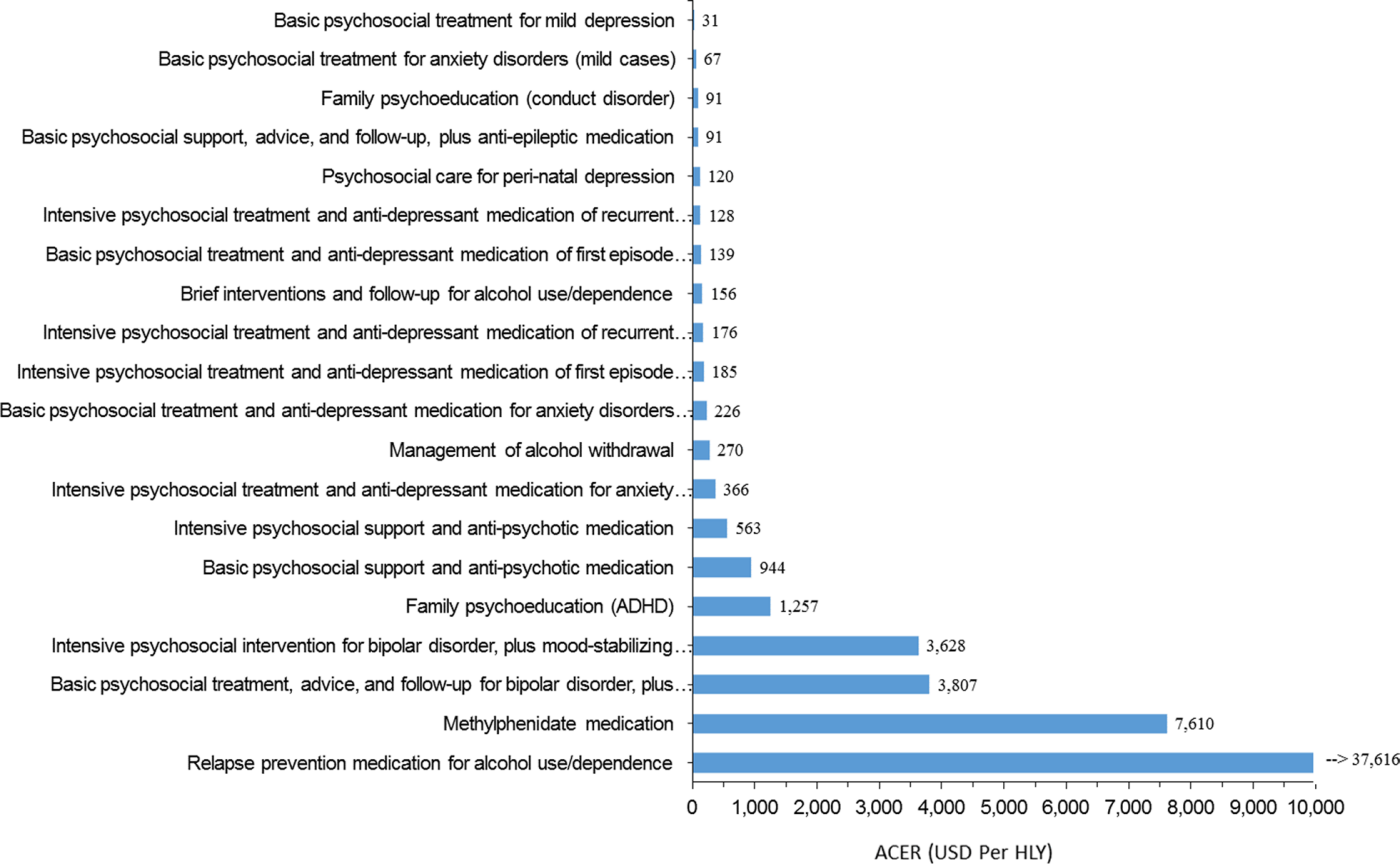
ACERs for mental health interventions (Trimmed at US$10,000)

In this study, we examined 20 mental health interventions. The provision of basic psychosocial treatment for mild depression is the most cost-effective intervention, with an ACER of US$31 per HLY, and basic psychosocial support for mild cases of anxiety disorder is the second most cost-effective (ACER = 67 USD/HLY). In the mental health intervention category, relapse prevention medication for alcohol use/dependence is the least cost-effective intervention, costing US$37,616 per HLY (Fig. 6).

### Cost effectiveness of chronic respiratory disease interventions

**Fig. 7:**
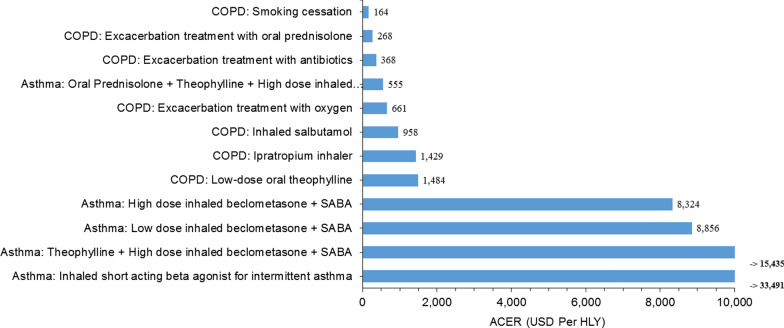
ACERs for chronic respiratory disease interventions (Trimmed at US$10,000)

We examined 12 interventions under the chronic respiratory disease category, and the provision of smoking cessation interventions to prevent chronic obstructive pulmonary disease (COPD) is the most cost effective (ACER = 164 USD/HLY). The provision of an inhaled, short-acting beta-agonist for intermittent asthma is the least cost-effective in this category, with an ACER of US$15,440 per HLY (Fig. 7).

## Discussion

This analysis aimed to provide input for the revision of Ethiopia’s EHSP, which used seven predefined and pre-agreed criteria, one being the cost effectiveness of interventions [7]. Our analysis encompasses a comprehensive range of health interventions, including preventive, promotive, curative and policy interventions. Of the interventions analysed in this study, a large majority (75%) have ACERs of less than US$1000, and 36% have ACERs below US$100.

Cost-effectiveness analysis is an increasingly important prioritisation tool. The cost-effectiveness evidence for redefining Ethiopia’s EHSP was generated in three ways: by contextualising CEA evidence from other studies using transferability criteria, by using expert opinion for multisectoral interventions and by using the WHO GCEA tool [7]. Using this tool, we provide cost-effectiveness evidence for 159 relevant health interventions for EHSP revision in Ethiopia. We believe that other low-income countries in Africa can also generate these pieces of evidence within a relatively short time and at an affordable cost compared with individual economic evaluation studies.

We provide cost-effectiveness evidence for 77 interventions on RMNCH and infectious disease (e.g., HIV, TB, nutrition, malaria and WASH) and for 82 interventions on NCDs. In general, a majority of the interventions have relatively low ACERs of less than US$1000 per HLY gained. However, when we disaggregate the finding by programme area, the results are mixed. While a vast majority (95%) of RMNCH and infectious disease interventions have an ACER of less than US$1000 per HLY, a substantial proportion (44%) of NCD interventions have an ACER of higher than US$1000 per HLY. In general, findings from our study are consistent with findings of other country specific studies in Ethiopia, Zimbabwe, Mexico [8–10, 30–32], or other regional and global estimates [33–38]. However, head to head comparison of the ACERs and further examination of cost, effectiveness, and its driving factors remain a priority for additional research.

Family planning interventions, for example, are the most cost effective in this study, with ACERs of less than US$1 per HLY gained. This very low ACER may be partly explained by the fact that the model accounts for a reduction in unplanned pregnancies and an associated reduction in maternal mortality. Most of the interventions targeting infectious diseases were cost effective, with an ACER of less than US$500 per HLY. For example, we evaluated four HIV/AIDS interventions, and they all, except the provision of cotrimoxazole for children, have an ACER of less than US$100 per HLY gained. The relatively low ACER in this study may partly reflect the decrement of the price of ART drugs as is shown in several recent studies [39, 40].

Addressing maternal, neonatal and child health issues is a top priority of the Ethiopian Ministry of Health (MoH) [7]. In our study, the majority of the interventions on RMNCH were very cost effective, with an ACER value of less than US$200 per HLY gained. This finding is in line with that of Memirie et al. in a CEA examining the cost effectiveness of 13 maternal and neonatal health (MNH) interventions in Ethiopia. Although not a GCEA and therefore not directly comparable, that study found that 12 of 13 MNH interventions had an incremental cost-effectiveness ratio of less than US$400 per HLY [9].

Most of the preventive NCD policy interventions have a lower cost-effectiveness ratio than the treatment NCD interventions. A substantial proportion (44%) of NCD interventions have an ACER of greater than US$1000 per HLY. This relatively high ACER may reflect the fact that the treatment cost for chronic NCD is higher and the treatment effectiveness lower than for the other interventions. This is particularly consistent with findings from a comprehensive, but relatively old study, examining 101 NCD interventions in Mexico. The study find similar variations among NCD policy interventions and NCD treatment interventions as we do [30].

### Strengths and limitations

By applying the GCEA approach, it is possible to evaluate whether the current mix of interventions is efficient and whether proposed new interventions are appropriate. Therefore, GCEA is a more appropriate approach than a marginal analysis for conducting a sector-wide cost-effectiveness analysis of interventions [14, 15]. In this study, which included 159 interventions from diverse programme areas, we conducted a sector-wide cost-effectiveness analysis. Although this study covers a substantial number of crucial interventions, it did not attempt to analyse all interventions in the Ethiopian health sector. We believe, however, that our findings can be used as benchmarks for making better-informed expert judgements on other interventions that could not be analysed in such a standardised way.

WHO-CHOICE GCEA tool is important tool for sector-wide analysis of cost-effectiveness of wider range of interventions for priority setting. A primary advantage of the WHO-CHOICE GCEA tool is the ability to compare many interventions at the same time based on the same assumptions on cost, disease epidemiology and other key health system parameters (e.g., human resource, financing, and infrastructure). When health system plans and strategies are designed, we should evaluate and compare the costs and outcomes of combinations of interventions. However, a barrier to conducting economic evaluation studies is that they are time consuming and demand large amounts of local data and local technical expertise. We believe that this study demonstrates that the existing platform, with a large support team and substantial commitment, makes such an extensive and comprehensive evaluation possible.

Our work has other limitations. First, in this study, we used the health system perspective. In Ethiopia, one-third of the total health care cost is covered by the out-of-pocket expenditure of individuals [19], which can influence individuals’ choices in accessing health care delivery. The choice of perspective should also be taken into consideration when interpreting the results. Second, in this GCEA study, we applied data from diverse sources to model the health impact of interventions and costs. Of course, modelling is inevitably an imperfect representation of reality, and, therefore, robust uncertainty analysis would to some extent alleviate this challenge. However, because of the vast number of interventions included in this analysis, we did not include a sensitivity analysis. Therefore, as the software expands, future GCEA analysis of this kind should integrate a sensitivity analysis of at least some of the critical drivers of costs and health impacts.

A third limitation of this study is the use of DALYs for estimating disease burden and health benefit. Critics of DALY argue that the measure itself has limitations [41, 42]. Using DALYs tends to underrepresent or overestimate the value of interventions (such as palliative care and family planning) with outcomes that are not readily measured in this metric as well as interventions in nutrition for which the outcomes are improved cognition rather than improved health [43]. This is a real limitation that was taken seriously in the revision of Ethiopia’s EHSP. For these interventions, we also relied on the expanded EHSP process with user involvement and expert judgements. Furthermore, criteria other than cost effectiveness, such as equity, financial risk protection, budget impact and public concern are also important for defining the EHSP [3]. A fourth limitation of this study is that the models used do not capture full health benefits. The most striking example is the LiST model which mainly considers mortality outcomes. Future analysis should also account for health benefits from RMNCH interventions that avert non-fatal conditions.

Additionally, there are gaps in the available evidence on the cost of interventions, which can be closed only by conducting substantially more research in developing countries. Therefore, we recommend a concerted effort to establish country-level cost databases. This could be combined with capacity building through the training of researchers to generate such evidence.

## Conclusion

Through the process described above, we calculated country-specific CEA values which were required to inform the decisions around which interventions to provide under Ethiopia’s essential health service package (EHSP). The present study shows that several potential cost-effective interventions are available in all program areas that could substantially reduce Ethiopia’s disease burden if scaled up.

## Supplementary Information

**Additional file 1.** Cost, health effect and ACER for 159 health intervention, 2019.

## Data Availability

The datasets supporting the conclusions of this is fully available in the article.

## Abbreviations

ACERs: Average cost effectiveness ratios
AIM: AIDS Impact Module
ART: Anti-retroviral therapy
CEA: Cost-effectiveness threshold
CHOICE: Choosing Interventions that are Cost Effective
COVID-19: Coronavirus disease of 2019
CMNNDs: Communicable, maternal, neonatal, and nutritional disorders
DALY: Disability-adjusted life year
EHSP: Essential health services package
ETB: Ethiopian Birr
GCEA: Generalized cost effectiveness analysis
GDP: Gross Domestic Product
COPD: Chronic obstructive pulmonary disease
HLYs: Health life years
IPT: Intermittent Preventive Therapy
LiST: Lives Saved Tool
LLINs: Long-Lasting Insecticidal Nets
MDR-TB: Multi-drug-resistance tuberculosis
MNH: Maternal and neonatal health
MoH: Ministry of Health
MSH: Management Sciences for Health
NCDs: Non-communicable diseases
PMTCT: Prevention of Mother to Child Transmission
RMNCH: Reproductive maternal neonatal and child health
TB: Tuberculosis
USDs: US Dollars
VIA: Visual inspection with acetic acid
WASH: Water sanitation and hygiene
WHO: World Health Organization

## References

[1] Glassman A, Giedion U, Smith PC (2017). What’s in, what’s out: designing benefits for Universal Health Coverage.

[2] Chalkidou K, Glassman A, Marten R, Vega J, Teerawattananon Y, Tritasavit N, Gyansa-Lutterodt M, Seiter A, Kieny MP, Hofman K, Culyer AJ (2016). Priority-setting for achieving universal health coverage. Bull World Health Organ.

[3] WHO Consultative Group on Equity and Universal Health Coverage: Making Fair Choices on the Path to Universal Health Coverage. Final Report of the WHO Consultative Group on Equity and Universal Health Coverage. Geneva: World Health Organization; 2014.

[4] Youngkong S, Kapiriri L, Baltussen R (2009). Setting priorities for health interventions in developing countries: a review of empirical studies. Trop Med Int Health.

[5] Mathes T, Jacobs E, Morfeld JC, Pieper D (2013). Methods of international health technology assessment agencies for economic evaluations–a comparative analysis. BMC Health Serv Res.

[6] Wiseman V, Mitton C, Doyle-Waters MM, Drake T, Conteh L, Newall AT, Onwujekwe O, Jan S (2016). Using economic evidence to set healthcare priorities in low-income and lower-middle-income countries: a systematic review of methodological frameworks. Health Econ.

[7] Federal Ministry of Health of Ethiopia (2019). Essential health services package of Ethiopia 2019.

[8] Hailu A, Lindtjorn B, Deressa W, Gari T, Loha E, Robberstad B (2018). Cost-effectiveness of a combined intervention of long lasting insecticidal nets and indoor residual spraying compared with each intervention alone for malaria prevention in Ethiopia. Cost Eff Resour Alloc.

[9] Memirie ST, Tolla MT, Desalegn D, Hailemariam M, Norheim OF, Verguet S, Johansson KA (2019). A cost-effectiveness analysis of maternal and neonatal health interventions in Ethiopia. Health Policy Plan.

[10] Strand KB, Chisholm D, Fekadu A, Johansson KA (2016). Scaling-up essential neuropsychiatric services in Ethiopia: a cost-effectiveness analysis. Health Policy Plan.

[11] Tolla MT, Norheim OF, Memirie ST, Abdisa SG, Ababulgu A, Jerene D, Bertram M, Strand K, Verguet S, Johansson KA (2016). Prevention and treatment of cardiovascular disease in Ethiopia: a cost-effectiveness analysis. Cost Eff Resour Alloc.

[12] Drummond M (2015). Methods for the economic evaluation of health care programmes.

[13] Edejer TT-T, Baltussen RM, Adam T, Hutubessy R, Acharaya A, Evans DB, Murray CJL (2003). Making choices in health: WHO guide to cost effectiveness analysis.

[14] Hutubessy R, Chisholm D, Edejer TT (2003). Generalized cost-effectiveness analysis for national-level priority-setting in the health sector. Cost Eff Resour Alloc.

[15] Murray CJ, Evans DB, Acharya A, Baltussen RM (2000). Development of WHO guidelines on generalized cost-effectiveness analysis. Health Econ.

[16] World Health Organization: Global Health Observatory Data Repository/World Health Statistics 2018.

[17] Ethiopia profile http://www.healthdata.org/ethiopia.

[18] Ethiopia: GDP per capita, current prices U.S. dollars per capita https://www.imf.org/external/datamapper/NGDPDPC@WEO/ETH.

[19] Federal Ministry of Health of Ethiopia (2019). National Health Accounts 2016/17.

[20] World Health Organization: Methods for the economic evaluation of health care interventions for priority setting in the health system: an update from WHOCHOICE. 2019.

[21] Lauer JA, Rohrich K, Wirth H, Charette C, Gribble S, Murray CJ (2003). PopMod: a longitudinal population model with two interacting disease states. Cost Eff Resour Alloc.

[22] Avenir Health: OneHealth Tool. https://www.avenirhealth.org/software-onehealth.php.

[23] Sanders R (2016). OneHealth tool intervention assumptions.

[24] Bertram MY, Stenberg K, Brindley C, Li J, Serje J, Watts R, Edejer TT (2017). Disease control programme support costs: an update of WHO-CHOICE methodology, price databases and quantity assumptions. Cost Eff Resour Alloc.

[25] Stenberg K, Lauer JA, Gkountouras G, Fitzpatrick C, Stanciole A (2018). Econometric estimation of WHO-CHOICE country-specific costs for inpatient and outpatient health service delivery. Cost Eff Resour Alloc.

[26] Gray A (2011). Applied methods of cost-effectiveness analysis in health care.

[27] Drummond MF, Sculpher MJ, Claxton K, Stoddart GL, Torrance GW (2015). Methods for the economic evaluation of health care programmes.

[28] World Health Organization (2001). Macroeconomics and health: investing in health for economic development report of the commission on macroeconomics and health.

[29] Woods B, Revill P, Sculpher M, Claxton K (2016). Country-level cost-effectiveness thresholds: initial estimates and the need for further research. Value Health.

[30] Salomon JA, Carvalho N, Gutierrez-Delgado C, Orozco R, Mancuso A, Hogan DR, Lee D, Murakami Y, Sridharan L, Medina-Mora ME, Gonzalez-Pier E (2012). Intervention strategies to reduce the burden of non-communicable diseases in Mexico: cost effectiveness analysis. BMJ.

[31] Hansen KS, Chapman G (2008). Setting priorities for the health care sector in Zimbabwe using cost-effectiveness analysis and estimates of the burden of disease. Cost Eff Resour Alloc.

[32] Horton S, Levin C, Black RE, Laxminarayan R, Temmerman M, Walker N (2016). Cost-effectiveness of interventions for reproductive, maternal, neonatal, and child health. Reproductive, maternal, newborn, and child health: disease control priorities.

[33] Ralaidovy AH, Gopalappa C, Ilbawi A, Pretorius C, Lauer JA (2018). Cost-effective interventions for breast cancer, cervical cancer, and colorectal cancer: new results from WHO-CHOICE. Cost Eff Resour Alloc.

[34] Adam T, Lim SS, Mehta S, Bhutta ZA, Fogstad H, Mathai M, Zupan J, Darmstadt GL (2005). Cost effectiveness analysis of strategies for maternal and neonatal health in developing countries. BMJ.

[35] Hogan DR, Baltussen R, Hayashi C, Lauer JA, Salomon JA (2005). Cost effectiveness analysis of strategies to combat HIV/AIDS in developing countries. BMJ.

[36] Baltussen R, Floyd K, Dye C (2005). Cost effectiveness analysis of strategies for tuberculosis control in developing countries. BMJ.

[37] Morel CM, Lauer JA, Evans DB (2005). Cost effectiveness analysis of strategies to combat malaria in developing countries. BMJ.

[38] World Health Organization (2017). Best buys and other recommended interventions for the prevention and control of noncommunicable diseases.

[39] Forsythe SS, McGreevey W, Whiteside A, Shah M, Cohen J, Hecht R, Bollinger LA, Kinghorn A (2019). Twenty years of antiretroviral therapy for people living with HIV: global costs, health achievements, economic benefits. Health Aff.

[40] Zegeye EA, Mbonigaba J, Kaye S, Johns B (2019). Assessing the cost of providing a prevention of mother-to-child transmission of HIV/AIDS service in Ethiopia: urban-rural health facilities setting. BMC Health Serv Res.

[41] Gold MR, Stevenson D, Fryback DG (2002). HALYS and QALYS and DALYS, Oh My: similarities and differences in summary measures of population Health. Annu Rev Public Health.

[42] Anand S, Hanson K (1997). Disability-adjusted life years: a critical review. J Health Econ.

[43] Eyal N, Hurst SA, Murray CJL, Schroeder SA, Wikler D, Eyal N, Hurst SA, Murray CJL, Schroeder SA, Wikler D (2020). Measuring the global burden of disease: philosophical dimensions. Population level bioethics series.

