# Supplementary material for: Generalised cost-effectiveness analysis of 159 health interventions for the Ethiopian essential health service package
Source: Cost Eff Resour Alloc. 2021 Jan 6;19:2. doi: 10.1186/s12962-020-00255-3 (PMC7787224; doi:10.1186/s12962-020-00255-3)
Supplement: Supplementary file 1 — Additional file 1. Cost, health effect and ACER for 159 health intervention, 2019. [file 12962_2020_255_MOESM1_ESM.pdf]

Additional file 1. Cost, health effect and ACER for 159 health intervention, 2019

S.No	Program	Intervention	Total cost (100 year)	Total HLY (100 year)	ACER
1	RMNCH	Pneumonia treatment with antibiotics + Zink supplementation	16,334,329,027	78,397,062	208.4
2	RMNCH	Skilled assistance for normal delivery	2,530,693,141	186,433,911	13.6
3	RMNCH	Preventing and managing unplanned pregnancy	2,692,042,674	6,505,365,924	0.4
4	RMNCH	Comprehensive antenatal care	4,994,559,942	25,730,716	194.1
5	RMNCH	Skilled assistance for normal delivery (including neonatal resuscitation)	1,928,413,000	149,210,690	12.9
6	RMNCH	Skilled assistance for normal delivery + family planning	3,230,785,619	6,818,691,861	0.5
7	RMNCH	Skilled delivery for management of complications	4,234,342,197	430,687,717	9.8
8	RMNCH	Skilled delivery + management of complications + family planning	4,204,818,503	6,960,176,453	0.6
9	RMNCH	New-born complications at referral level	1,418,692,491	106,628,451	13.3
10	RMNCH	Community-based new-born and childcare	9,038,945,306	238,745,780	37.9
11	RMNCH	Infant and young child feeding	1,973,967,713	41,905,462	47.1
12	RMNCH	Routine EPI + additional vaccines (rotavirus, pneumococcal, hep B)	5,836,793,286	85,274,632	68.5
13	RMNCH	Primary level integrated management of the sick child (includes link to the community)	9,446,454,994	152,927,064	61.8
14	RMNCH	Family planning	2,585,122,410	6,146,941,266	0.4
15	RMNCH	Safe abortion	308,093,915	2,136,941	144.2
16	RMNCH	Post-abortion case management	195,004,722	1,750,142	111.4
17	RMNCH	Ectopic case management	303,923,860	443,585	685.2
18	RMNCH	Tetanus toxoid (pregnant women)	748,251,829	49,437,522	15.1
19	RMNCH	Syphilis detection and treatment (pregnant women)	355,739,050	6,046,651	58.8
20	RMNCH	Hypertensive disorder case management	213,594,777	2,210,246	96.6
21	RMNCH	Management of pre-eclampsia (magnesium sulphate)	424,128,445	8,347,632	50.8
22	RMNCH	Labour and delivery management	1,329,157,840	275,647,049	4.8
23	RMNCH	Active management of the third stage of labour	253,739,530	2,202,168	115.2
24	RMNCH	Management of eclampsia (magnesium sulphate)	258,038,489	2,000,640	129.0
25	RMNCH	Neonatal resuscitation (institutional)	203,618,525	35,997,997	5.7

26	RMNCH	Kangaroo mother care	584,927,929	26,025,214	22.5
27	RMNCH	Clean practices and immediate essential new-born care (home)	537,017,585	23,879,689	22.5
28	RMNCH	Antibiotics for premature rupture of membrane (pPRoM)	288,047,059	8,699,604	33.1
29	RMNCH	Induction of labour (beyond 41 weeks)	197,058,028	8,681,269	22.7
30	RMNCH	Maternal sepsis case management	724,763,890	2,125,295	341.0
31	RMNCH	New-born sepsis – full supportive care	798,757,637	77,766,357	10.3
32	RMNCH	New-born sepsis – injectable antibiotics	342,281,399	63,656,619	5.4
33	RMNCH	Clean postnatal practices	932,099,982	29,899,387	31.2
34	RMNCH	Chlorhexidine	292,245,501	14,743,167	19.8
35	RMNCH	Zinc supplementation	14,543,051,384	9,138,667	1,591.4
36	RMNCH	Oral rehydration salt (ORS)	2,124,463,258	60,080,074	35.4
37	RMNCH	Zinc (diarrhoea treatment)	3,441,519,134	13,232,917	260.1
38	RMNCH	Antibiotics for treatment of dysentery	962,682,242	5,659,880	170.1
39	RMNCH	Pneumonia treatment (children)	1,977,589,800	72,540,746	27.3
41	RMNCH	Rotavirus vaccine	1,696,748,830	7,586,795	223.7
42	RMNCH	Measles vaccine	541,533,638	42,584,395	12.7
43	RMNCH	DPT vaccination	1,162,135,191	23,459,045	49.5
44	RMNCH	Hib vaccine	3,285,116,860	67,728,499	48.5
45	RMNCH	Pneumococcal vaccine	3,020,020,041	35,293,570	85.6
46	RMNCH	Intermittent iron-folic acid supplementation (menstruating women where anaemia is a public health problem)	2,783,956,085	3,730,930	746.2
47	RMNCH	Daily iron and folic acid supplementation (pregnant women)	920,362,816	1,328,307	692.9
48	RMNCH	Calcium supplementation for prevention and treatment of pre-eclampsia and eclampsia	3,726,480,194	6,422,349	580.2
49	RMNCH	Nutritional care and support for pregnant and lactating women in emergencies	1,370,290,264	43,872,169	31.2
50	RMNCH	Breastfeeding counselling and support	718,041,292	19,307,445	37.2
51	RMNCH	Complementary feeding counselling and support	564,812,972	7,802,229	72.4
52	RMNCH	Vitamin A supplementation in infants and children 6–59 months	1,009,514,923	15,104,781	66.8
53	RMNCH	Management of severe malnutrition (children)	1,480,455,119	14,631,168	101.2
54	RMNCH	Management of moderate acute malnutrition (children)	1,031,799,721	30,853,981	33.4
55	HIV/AIDS	Prevention of Mother to Child Transmission of HIV (PMTCT)	220,108,921	3,630,711	60.6
56	HIV/AIDS	ART (first-line treatment) for men	227,796,548	6,770,988	33.6
57	HIV/AIDS	ART (first-line treatment) for women	227,796,548	17,651,995	12.9

58	HIV/AIDS	Cotrimoxazole for children	216,363,590	536,452	403.3
59	HIV/AIDS	Paediatric ART	342,989,847	17385351	19.7
60	TB	Treatment + detection (smear + Xpert) + drug sensitivity analysis	44,640,122	340340	131.2
61	TB	Treatment + detection (smear + Xpert) + drug sensitivity analysis & ART prioritisation for TB cases	47,084,805	342060	137.7
62	TB	Treatment + detection (smear + Xpert) + drug sensitivity analysis & ART prioritisation for TB cases & preventive therapy & preventive therapy for children	55,726,027	342578	162.7
63	TB	Treatment + detection (smear + Xpert) + drug sensitivity analysis & preventive therapy	50,028,015	340542	146.9
64	TB	Treatment + detection (smear + Xpert) + drug sensitivity analysis & preventive therapy for children	47,962,710	340536	140.8
65	TB	Treatment + detection (smear generally and culture for MDR) + drug sensitivity analysis	43,582,993	337276	129.2
66	TB	Treatment + detection (smear generally and culture for MDR) + drug sensitivity analysis & ART prioritisation for TB cases	46,710,175	339329	137.7
67	TB	Treatment + detection (smear generally and culture for MDR) + drug sensitivity analysis & ART prioritisation for TB cases & preventive therapy for children	55,061,921	340401	161.8
68	TB	Treatment + detection (smear generally and culture for MDR) + drug sensitivity analysis & preventive therapy	49,403,233	337657	146.3
69	TB	Treatment + detection (smear generally and culture for MDR) + drug sensitivity analysis & preventive therapy for children	46,260,546	337766	137.0
70	Malaria	Insecticide treated materials	392904105	4961118	79.2
71	Malaria	Indoor residual spraying	197112989	4961118	39.7
72	Malaria	IPT (pregnant women)	302685897	231000	1,310.3
73	Malaria	Treatment of malaria (pregnant women)	223573250	152244	1,468.5
40	RMNCH	Malaria treatment (children 0–4)	20,707,464,309	7,103,167	2,915.2
74	NCD policy interventions	Physical activity + obesity reduction	497,709,465	77,987	6,382.0
75	NCD policy interventions	Tobacco: protect people from tobacco smoke	197,023,733	849,840	231.8
76	NCD policy interventions	Tobacco: warn about danger (warning labels)	348,952,855	849,840	410.6
77	NCD policy interventions	Tobacco: warn about danger (mass media campaign)	355,762,310	690,328	515.4
78	NCD policy interventions	Tobacco: enforce bans on tobacco advertising	208,886,108	1,991,147	104.9
79	NCD policy interventions	Tobacco: enforce youth access restriction	355,762,310	205,858	1,728.2
80	NCD policy interventions	Hazardous alcohol use: enforce restrictions on availability of retailed alcohol	207,765,195	47,465	4,377.2
81	NCD policy interventions	Hazardous alcohol use: enforce restrictions on alcohol advertising	207,765,195	22,795	9,114.6

82	NCD policy interventions	Physical inactivity: awareness campaigns to encourage increased physical activity	355,762,310	77,987	4,561.8
83	NCD policy interventions	Sodium: harness industry for reformulation	207,765,195	7,868,153	26.4
84	NCD policy interventions	Sodium: adopt standards (front-of-pack labelling)	207,765,195	4,961,006	41.9
85	NCD policy interventions	Sodium: knowledge (education and communication)	355,762,310	1,066,921	333.4
86	NCD policy interventions	Sodium: environment (salt reduction strategies in community-based eating spaces)	243,296,086	1,406,571	173.0
87	NCD policy interventions	Offer to help cease tobacco use: brief intervention	197,023,733	424,646	464.0
88	NCD policy interventions	Screening and brief intervention for hazardous and harmful alcohol use	197,023,733	340,403	578.8
89	NCD policy interventions	Physical inactivity: brief advice as part of routine care	197,023,733	647,584	304.2
90	Breast cancer	Clinical breast exam + treatment, stage 1	2,643,068,127	1,777,450	1,487.0
91	Breast cancer	Clinical breast exam + treatment, stage 2	2,776,430,900	2,260,258	1,228.4
92	Breast cancer	Clinical breast exam + treatment, stage 3	2,807,624,806	1,774,576	1,582.1
93	Breast cancer	Clinical breast exam + treatment, stage 4	2,716,615,426	1,427,863	1,902.6
94	Breast cancer	Screening: clinical breast exam	2,568,713,997	1,166,025	2,203.0
95	Breast cancer	Screening: mammography	1,661,513,699	272,205	6,103.9
96	Breast cancer	Breast cancer treatment, stage 2	346,901,998	946,750	366.4
97	Breast cancer	Breast cancer treatment, stage 3	434,535,125	681,809	637.3
98	Breast cancer	Breast cancer treatment, stage 4	345,963,279	335,321	1,031.7
99	Breast cancer	Basic palliative care for breast cancer	271,393,565	53,950	5,030.4
100	Cervical cancer	HPV DNA and cryotherapy	1,144,822,100	18,590,572	61.6
101	Cervical cancer	VIA and cryotherapy	712,825,781	19,548,374	36.5
102	Cervical cancer	PAP smear and cryotherapy	691,635,007	19,253,223	35.9
103	Cervical cancer	HPV DNA test	1,110,069,686	18,431,248	60.2
104	Cervical cancer	Visual inspection with acetic acid (VIA)	678,083,133	19,548,374	34.7
105	Cervical cancer	Papanicolaou test (Pap smear)	656,892,359	19,253,223	34.1
106	Cervical cancer	HPV DNA + VIA	2,191,347,190	18,923,081	115.8
107	Cervical cancer	HPV DNA + Pap smear	2,065,785,596	18,648,847	110.8
108	Cervical cancer	Cervical cancer treatment, stage 1	197,023,733	726,991	271.0
109	Cervical cancer	Cervical cancer treatment, stage 2	707,229,588	1,125,956	628.1
110	Cervical cancer	Cervical cancer treatment, stage 3	999,413,857	865,909	1,154.2
111	Cervical cancer	Cervical cancer treatment, stage 4	1,136,333,690	510,268	2,226.9
112	Cervical cancer	Basic palliative care for cervical cancer	312,657,375	47,853	6,533.7
113	Colorectal cancer	Colonoscopy + colorectal Ca treatment, stage 1	7,526,238,218	1,381,807	5,446.7

114	Colorectal cancer	Colonoscopy + colorectal Ca treatment, stage 2	7,548,699,303	1,624,657	4,646.3
115	Colorectal cancer	Colonoscopy + colorectal Ca treatment, stage 3	7,694,679,772	1,725,395	4,459.7
116	Colorectal cancer	Colonoscopy + colorectal Ca treatment, stage 4	7,528,376,687	1,385,034	5,435.5
117	Colorectal cancer	Screening: Faecal occult blood testing	8,197,942,546	1,463,347	5,602.2
118	Colorectal cancer	Screening: Sigmoidoscopy	3,218,784,997	1,291,163	2,492.9
119	Colorectal cancer	Screening: Colonoscopy	7,486,841,501	1,381,807	5,418.2
120	Colorectal cancer	Colorectal cancer treatment, stage 2	233,065,249	297,804	782.6
121	Colorectal cancer	Colorectal cancer treatment, stage 3	441,700,031	443,463	996.0
122	Colorectal cancer	Colorectal cancer treatment, stage 4	203,914,155	4,208	48,460.7
123	Colorectal cancer	Basic palliative care for colorectal cancer	201,034,208	46	4,390,959.0
124	Respiratory disease	Asthma: inhaled short acting beta-agonist for intermittent asthma	1,278,008,012	38,159	33,491.9
125	Respiratory disease	Asthma: low-dose inhaled beclometasone + SABA	5,611,860,132	633,679	8,856.0
126	Respiratory disease	Asthma: high-dose inhaled beclometasone + SABA	6,697,801,912	804,626	8,324.1
127	Respiratory disease	Asthma: theophylline + high-dose inhaled beclometasone + SABA	11,110,907,521	719,635	15,439.6
128	Respiratory disease	Asthma: oral prednisolone + theophylline + high-dose inhaled beclometasone + SABA	615,943,800	1,109,263	555.3
129	Respiratory disease	COPD: smoking cessation	423,685,328	2,584,364	163.9
130	Respiratory disease	COPD: inhaled salbutamol	9,531,825,169	9,948,246	958.1
131	Respiratory disease	COPD: low-dose oral theophylline	5,513,831,736	3,714,578	1,484.4
132	Respiratory disease	COPD: ipratropium inhaler	4,580,700,867	3,206,483	1,428.6
133	Respiratory disease	COPD: exacerbation treatment with antibiotics	555,224,249	1,510,580	367.6
134	Respiratory disease	COPD: exacerbation treatment with oral prednisolone	730,031,926	2,728,661	267.5
135	Respiratory disease	COPD: exacerbation treatment with oxygen	2,905,335,246	4,398,446	660.5
136	Mental health	Basic psychosocial treatment for anxiety disorders (mild cases)	358,637,743	5,343,575	67.1
137	Mental health	Basic psychosocial treatment and anti-depressant medication for anxiety disorders (moderate–severe cases)	1,062,921,311	4,704,980	225.9
138	Mental health	Intensive psychosocial treatment and anti-depressant medication for anxiety disorders (moderate–severe cases)	2,042,727,108	5,579,910	366.1
139	Mental health	Basic psychosocial treatment for mild depression	197,023,733	6,352,788	31.0
140	Mental health	Basic psychosocial treatment and anti-depressant medication of first episode, moderate–severe cases	576,831,856	4,153,676	138.9
141	Mental health	Intensive psychosocial treatment and anti-depressant medication of first episode, moderate–severe cases	953,448,079	5,156,457	184.9

142	Mental health	Intensive psychosocial treatment and anti-depressant medication of recurrent moderate–severe cases on an episodic basis	2,033,893,515	11,536,064	176.3
143	Mental health	Intensive psychosocial treatment and anti-depressant medication of recurrent moderate–severe cases on a maintenance basis	2,750,850,299	21,491,348	128.0
144	Mental health	Psychosocial care for perinatal depression	197,023,733	1,637,021	120.4
145	Mental health	Basic psychosocial support and anti-psychotic medication	1,106,762,360	1,171,847	944.5
146	Mental health	Intensive psychosocial support and anti-psychotic medication	1,120,930,627	1,992,140	562.7
147	Mental health	Basic psychosocial treatment, advice and follow-up for bipolar disorder + mood-stabilising medication	6,768,181,998	1,777,842	3,807.0
148	Mental health	Intensive psychosocial intervention for bipolar disorder + mood-stabilising medication	7,525,974,010	2,074,149	3,628.5
149	Mental health	Basic psychosocial support, advice and follow-up + anti-epileptic medication	693,439,091	7,602,401	91.2
150	Mental health	Family psychoeducation (ADHD)	197,023,733	156,684	1,257.5
151	Mental health	Family psychoeducation (conduct disorder)	197,023,733	2,164,818	91.0
152	Mental health	Methylphenidate medication	547,720,758	71,977	7,609.7
153	Mental health	Brief interventions and follow-up for alcohol use/dependence	290,402,140	1,863,433	155.8
154	Mental health	Management of alcohol withdrawal	340,802,187	1,263,547	269.7
155	Mental health	Relapse prevention medication for alcohol use/dependence	70,095,872,008	1,863,433	37,616.5
156	WASH	Use of improved water source within 30 minutes	121,186,129	268,924	450.6
157	WASH	Use of water connection in the home	120,901,917	23,202,281	5.2
158	WASH	Improved excreta disposal (latrine/toilet)	121,002,008	6,592,710	18.4
159	WASH	Hand washing with soap	120,878,085	9,488,699	12.7
